# Screen Time, Nature, and Development: Baseline of the Randomized Controlled Study “Screen‐free till 3”

**DOI:** 10.1111/desc.13578

**Published:** 2024-10-23

**Authors:** Silke Schwarz, Hanno Krafft, Tobias Maurer, Silke Lange, Juliane Schemmer, Thomas Fischbach, Anke Emgenbroich, Sean Monks, Michael Hubmann, David Martin

**Affiliations:** ^1^ Faculty of Health, Department of Human Medicine Witten/Herdecke University Witten Germany; ^2^ Professional Association of Pediatricians and Adolescent Doctors (BVKJ) Cologne Germany; ^3^ BVKJ‐Service GmbH Cologne Germany; ^4^ Monks Ärzte‐im‐Netz GmbH Hamburg Germany; ^5^ University Clinic for Pediatric and Adolescent Medicine University of Tübingen Tübingen Germany

**Keywords:** children, CIUS, development, parents, randomized intervention study, screen media

## Abstract

In the first years of life, increased screen media use is presumably associated with health consequences and developmental impairments. “Screen‐free till 3” is a prospective Germany‐wide randomized intervention study, started in May 2022 with a duration of 3 years. In the intervention group, 2581 pediatric practices received stickers, which were systematically placed in the screening booklet of all children, along with advice to parents to keep children free from screens until the age of 3. A volunteer sample of 17,436 parents received an invitation to take part in the preinterventional questionnaire. The outcomes were parents' internet use (CIUS test), parental screen time in the presence of children, time of screen media in the background, and children's development. Four thousand twenty‐one parents answered the questionnaire. 16.7% of mothers and 31.0% of fathers reached the CIUS score of an internet‐related disorder. Parents whose children use screen media at an early age had significantly higher CIUS values on average (*M* = 4.07) than the parents of children who do not yet have any screen time (*p* < 0.001). Combined developmental characteristics show a negative correlation with parental screen time (*p* < 0.001). Time spent in nature was positively associated with development (*p* < 0.001). The evaluation of the survey shows that screen media is to a large extent used on a daily basis. The study confirms the assumption that high screen media use by parents is linked to higher screen media use by children and also has a negative impact on child development.

**Trial Registration**: Number: RKS00032258; https://drks.de/search/en/trial/DRKS00032258

**Abbreviations**
AAP: American Academy of Pediatrics
AWMF: Association of the Scientific Medical Societies (Arbeitsgemeinschaft der Wissenschaftlichen Medizinischen Fachgesellschaften)
BB3: Screen‐free till 3 (Bildschirmfrei bis 3)
BVKJ: Professional Association of Pediatricians and Adolescent Doctors (Berufsverband der Kinder‐ und Jugendärzt*innen)
BZgA: Federal Centre for Health Education in Germany (Bundezentrale für gesundheitliche Aufklärung)
CIUS: Compulsive Internet Use Scale
DAK: Statutory health insurance in Germany (DAK‐Gesundheit)
DZSKJ: German Center for Addiction Research in Childhood and Adolescence (Deutschen Zentrums für Suchtfragen des Kindes‐ und Jugendalters)
PSTC: Parent screen time in the presence of the child
EEG: Electroencephalography
Forsa: Market and opinion research institute in Germany
ICD‐1: 111th version of the International Statistical Classification of Diseases and Related Health Problems
KJÄ: Pediatricians and adolescent physicians
MPP app: “My pediatric practice” app

1

**Summary**

- Screen media is to a large extent used on a daily basis in young families
- High screen media use by parents is linked to higher screen media use by children
- High screen media use by parents and children might have a negative impact on child development

## 2 Background

The spread of the internet, mobile devices, and software developments, such as in the field of communication or computer games, have had an enormous impact on society. The rapid technological progress in this area in recent years has led to screen media being firmly integrated into everyday life. The competent use of screen media is nowadays highly relevant, especially with regard to later career advancement. A lack of access to or skills in using screen media can jeopardize social participation. In addition to the opportunities and barriers, screen media also harbor tangible dangers, which represent a foreseeable risk, especially for children. The effects of screen media on newborns, infants, and toddlers deserve more investigations, but overall negative consequences are emerging. Excessive screen use is associated with various health indicators in physical, behavioral, and psychosocial aspects (Hutton et al. 2020, 2022; Law et al. 2023; Li et al. 2020; Madigan et al. 2020; Schwarzer et al. 2022; Yang et al. 2020). The German national AWMF guideline on the prevention of dysregulated digital screen media use in childhood and adolescence recommends screen time restriction for the first 3 years of life and a maximum daily screen time of 30 min per day for 3 to 6‐year‐olds (Deutsche Gesellschaft für Kinder‐ und Jugendmedizin e.V. DGKJ 2023). A survey conducted in 2022 revealed an average screen time of 93 min per day for boys and 83 min per day for girls among 3‐ to 5‐year‐olds (Insights and Analytics SUPER RTL 2022). This means that screen media exposure, that is, the time spent actively and passively using screen media such as TV, smartphone, tablet, or computer, is currently well above the recommendations for children in Germany. The American Academy of Pediatrics (AAP) recommends that babies between 0 and 18 months do not use screen media at all, that toddlers between 18 and 24 months only have screen time with parental involvement, and that 2‐ to 5‐year‐olds should limit screen use to 1‐h per day with high‐quality programs (American Academy of Pediatrics 2023). Especially for a healthy development in the first 3 years of life, screen media exposure is correlated with severe developmental impairment. Early screen media exposure is associated with obesity (Biddle, García Bengoechea, and Wiesner 2017; Zhang et al. 2022), insulin resistance and type 2 diabetes mellitus (Nagata et al. 2023; Sina et al. 2021), sleep problems (Li et al. 2020) and myopia in preschoolers(Yang et al. 2020), delayed language development, language problems, math learning problems, writing and reading problems, structural differences in the brain (Hutton et al. 2022, 2020; Li et al. 2020; Madigan et al. 2020), fine motor and gross motor developmental deficits (Li et al. 2020), social–emotional delays, hyperactivity, inattention, aggressive and antisocial behavior and other behavioral problems (Li et al. 2020), altered cortical electroencephalography (EEG) activity associated with altered executive functions (Law et al. 2023), and altered mental imagery (Suggate and Martzog 2020). Some of these findings showed a dose‐dependent effect (Hutton et al. 2020; Law et al. 2023; Madigan et al. 2020). A recent study showed that increased screen time at 2 years of age was directly related to decreased communication skills and daily living skills at 4 years of age. The frequency of outdoor play mitigated this association (Sugiyama et al. 2023). Previous interventions to reduce screen time in the first years of life have shown inconsistent effects on children's screen time (Krafft et al. 2023). To reduce the use of screen media by toddlers and young families, the study “Screen‐free till 3” (in German “Bildschirmfrei bis 3,” BB3) was initiated in cooperation with the Professional Association of Pediatricians (BVKJ) as an outpatient care research study. The primary aim of the BB3 intervention is to ensure that children under the age of 3 are protected as much as possible from active or passive screen media exposure. This is achieved by pediatricians informing parents with the support of the “Screen‐free till 3” material. Secondarily, by reducing screen time, the screen media‐associated morbidity rates should be reduced. This paper reports on the baseline “Screen‐free till 3” intervention. The research question of this first evaluation of the “Screen‐free till 3” study is to obtain a baseline of the study participants with regard to their screen use behavior, their leisure activities, and the development of the children. The further research hypothesis, derived from the existing research literature, is that there is a negative correlation between screen use behavior and development.

## 3 Methods

The study includes all children born in 2022 in Germany and investigates child development and family behavior in relation to screen media use from the 6th month of life to the age of 3 years in the subset of those parents who had access to the BVKJ's “My Pediatric Practice” app (MPP app). The intervention and measurement dates correspond to statutory screening examinations in Germany at certain ages, whereby the present baseline data refer to the statutory screening examination at the age of 5 to 7 months.

### 3.1 Intervention

There was a two‐thirds randomization of all pediatric practices in Germany in favor of the intervention group. The intervention group (*n* = 2581 pediatric practices) was provided with the study materials; they received a “starter pack” by post, inviting them to participate in the study on a voluntary basis. With the starter pack, the practices in the intervention group received an information letter with an invitation to an online training course, a poster with a QR code for the waiting room, and 100 signal stickers with the motto “Screen‐free till 3.” The material can be seen on https://bildschirmfrei‐bis‐3.de/en/parents/. During the training, the pediatricians were instructed to systematically place the signal stickers, accompanied by a verbal message with positive emotions, in the official examination booklet of all children during the statutory examination at the age of 6 to 7 months, with a tolerance boundary of 5 to 8 weeks; this examination is called the “U5” examination. As the practices were recruited by cold calling, the study materials also included a reply postcard with which the practices could confirm their participation. Based on the response rate, the participation rate of the intervention practices was 39% (1009 practices).

**FIGURE 1:**
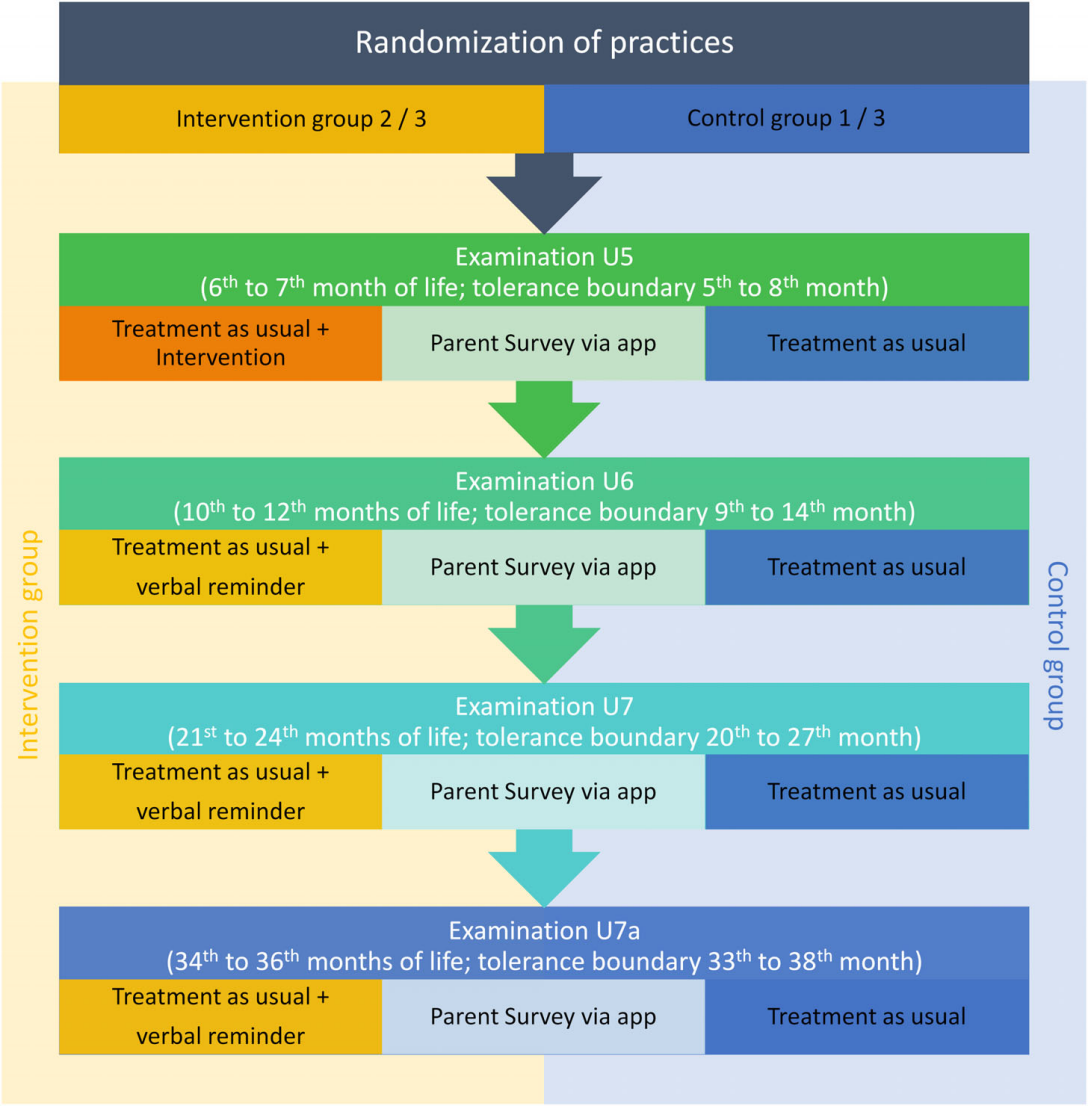
Flowchart of the study design.

The control group (*n* = 1295 practices) did not receive any study material and treated their patients according to the usual standard of practice (treatment as usual). The subsequent examinations (U6 at age 10–12 months, U7 at 21 to 24 months and U7a at 34 to 36 months) are carried out by all practices in accordance with standard care; the parents who used the MPP app are repeatedly invited to complete the survey on child development and media use in the family at the respective examination time (Figure 1).

### 3.2 Evaluation

The quantitative recording of the use of digital screen media in families and child development will be conducted over 3 years using the MPP app, which is currently offered by around 1191 practices of the approximately 3500 pediatric practices registered in Germany. Parents are invited to take part in the survey in the MPP app by push notifications before the screening examinations. The preinterventional questionnaire before the age of 5 to 7 months (U5) includes 16 questions on the parents' sociodemographics, 6 questions on the child's and parents' screen media use, 8 questions on the child's development, and 5 questions on the parents' internet use according to the short compulsive internet use scale (short‐CIUS) (Besser et al. 2017). In this article, we report on the preintervention results of the quantitative survey of the sociodemographic baseline, screen behavior, child development, and other possible influencing factors, including time in nature.

### 3.3 Statistical Analyses

The collected data were cleaned using the software R 4.3.1 (R Core Team 2023). The statistical calculations were carried out using SPSS V29 (IBM Corp., Armonk, NY, USA). The descriptive analysis of the data includes the absolute and relative frequencies of the variables, mean values, and medians, as well as Cronbach's alpha. The following statistical tests were also used for the analysis: Chi^2^ test to find correlations between two nominally scaled variables, Spearman's correlations for correlations between ordinal variables and Pearson's correlations for correlations between interval‐scaled variables. In addition, *T*‐tests were used to find differences between groups of metrically scaled variables. As there are clear outliers for some variables that should be deliberately included in the statistical calculation, the Mann–Whitney *U* test was chosen for the analysis as a method that cannot be distorted by outliers. No correction for multiple testing was performed, as such a correction would reduce the probability of alpha error but increase the probability of beta error. Since this is a first‐time study on the potential risks of screen time for children, it is crucial to consider the consequences. In this context, failing to recognize the effects of screen time could be seen as a greater issue than falsely confirming effects that do not exist. Furthermore, the study is exploratory in nature, and within a scientific framework, it seems more important to postulate effects that can be further examined by future research rather than dismissing them prematurely. Therefore, we prioritize accepting a higher alpha error over accepting a higher beta error.

The CIUS items were added together, and a CIUS scale was formed (range 0 to 20, Cronbach's *α* = 0.77). Scored using the recommended gender‐independent threshold of 7 or more points, at which the Short CIUS has a sensitivity of 0.95 and a specificity of 0.87 (Besser et al. 2017). The threshold value described indicates an internet‐related disorder.

## 4 Results

By the time all children born in 2022 were 7 months old, 4831 complete questionnaires from the preinterventional survey were answered. After checking the inclusion criteria and adjustment, 4021 data sets were included in the analysis. These data came from 651 practices throughout Germany. An overview of the demographic characteristics of the parents and children is shown in Table 1.

**TABLE 1:** Demographic characteristics of the participating parents and children.

*Parents in total N = 4021*	Absolute Frequency	Relative Frequency %^a^
Role of the participants		
Mothers	3820	95.0
Fathers	191	4.8
Foster parents	7	0.2
Other	3	0.1
Age of child (in months)		
5	2803	69.7
6	582	14.5
7	377	9.4
8	259	6.4
Gender of child		
Male	2071	51.5
female	1948	48.4
diverse	2	0
Who does the child live with?		
Both parents constantly	3881	96.5
Mother	121	3.0
Both parents alternating	8	0.2
Foster parents	6	0.1
Father	4	0.1
Other	1	0.0
Is your child looked after by a childminder/grandparent/relative, and so forth?		
No	3630	90.3
Yes	379	9.4
Mother's highest school‐leaving qualification		
University entrance qualification	2484	61.8
Intermediate secondary school leaving certificate	1291	32.1
Lower secondary school leaving certificate	221	5.5
Without a degree	25	0.6
Father's highest school‐leaving qualification		
University entrance qualification	1989	49.5
Intermediate secondary school leaving certificate	1407	35.0
Lower secondary school leaving certificate	565	14.1
Without a degree	60	1.5
Migration background/immigration		
No	3535	87.9
Yes	380	9.5
Not specified	106	2.6
Number of siblings		
0	2126	52.9
1	1389	34.6
2	385	9.6
3	73	1.8
4 or more	43	1.1
Age of the siblings		
0–3 (infants)	1140	45.9
4‐6 (preschool children)	646	26.1
7–10 (primary school children)	366	14.8
11–13 (prepuberty)	166	6.7
14–18 (puberty)	129	5.2
> 18 years	34	1.3
In what week of pregnancy was the birth?		
< 32	32	0.8
32–37	422	10.5
38–42	3526	87.7
> 42	6	0.1
Was the pregnancy a high‐risk pregnancy?		
No	2939	73.1
Yes	1070	26.6
Did your child receive hospital treatment after birth?		
No	3170	78.8
Yes	839	20.9
Does your child have any congenital diseases?		
No	3886	96.6
Yes	123	3.1
Are you satisfied with your child's development?		
Yes	3959	98.5
No	50	1.2
Is your child undergoing medical treatment?		
No	3769	93.7
Yes	240	6.0
Does your child have their own room?		
No, the child sleeps in the parents' bedroom	2874	71.5
Yes	1056	26.3
No, the child shares the room with brother/sister/siblings	79	2.0

^a^Missing 100%: no answer/do not know.

### 4.1 Screen Media Use

The number of minutes their child spends in front of screens per day was given as 0 min by 80.1% of parents, followed by up to 30 min by 16.7% of parents. In relation to the parents who completed the questionnaire, 53.5% of parents stated that their parental screen time in the presence of their child (PSTC) was up to 1‐h per day and 18.9% up to 2 h per day. 64.9% of parents stated that their child spent between half an hour and 2 h a day outdoors. The screen media usage behavior of children and parents can be found in Table 2.

**TABLE 2:** Screen media use of children and parents.

*Parents in total N = 4021*	*Absolute Frequency*	Relative Frequency %^a^
How many minutes a day does your child spend in front of screens? (active use)		
0	3222	80.1
1–5	284	7.1
6–15	231	5.7
16–30	158	3.9
31–60	69	1.7
61–120	36	0.9
More than 120	16	0.4
How many minutes a day do you spend with screens in front of your child? (PSTC)		
0	640	15.9
1–30	1113	27.7
31–60	1038	25.8
61–120	760	18.9
121–180	252	6.3
181–240	104	2.6
241–300	62	1.5
more than 300	45	1.1
How many minutes a day does your child spend in nature?		
0	31	0.8
1–30	226	5.3
31–60	1068	26.6
61–120	1539	38.3
121–180	607	15.1
181–240	284	7.1
241–300	127	3.2
more than 300	105	2.6

Abbreviation: PSTC, parent screen time in the presence of the child.

^a^Missing 100%: no answer/do not know.

### 4.2 Parental Internet Use

In the CIUS self‐test on problematic internet use, 10.4% of parents stated that they often or very often find it difficult to stop using the internet; 10.3% stated that they go online when they feel depressed. The mean CIUS score for mothers was 3.65 (0.19; 95% CI: 3.56–3.75). 16.7% (637) of the mothers reached the threshold value (≥ 7), which indicates an internet‐related disorder, that is, risky, harmful, or dependent internet use. For fathers, the mean value was 4.45 (0.16; 95% CI: 3.9–4.97), with 31.0% (59) reaching the threshold of concern. This is significantly higher than for the mothers. There is merely a very weak correlation between the mothers' school‐leaving qualification (*ρ* = 0.037, *p* = 0.018) and the achievement of the threshold value in the CIUS; the same applies to the fathers (*ρ* = 0.032, *p* = 0.040). The *t*‐test showed in the group comparison that the parents whose children already use screen media themselves had significantly higher CIUS values on average (*M* = 4.07) than the parents of children who do not yet have any screen time (*M* = 3.60) (*t*‐test (1170,231) = −3.846, *p* < 0.001). A problematic CIUS score of the parents (≥ 7) correlated only very weakly with the amount of active screen time of the children (*ρ* = 0.057, *p* < 0.001). The PSTC also correlated significantly with the parents' CIUS threshold (*ρ* = 0.148, *p* < 0.001). Of the children who had any screen time at all, 21.2% of parents exceed the CIUS threshold. Among children who have no screen time, only 16.4% of parents reach the threshold (*χ* ^2^(1) = 10.27, *p* < 0.001).

### 4.3 Screen Time and Child Development

**FIGURE 2:**
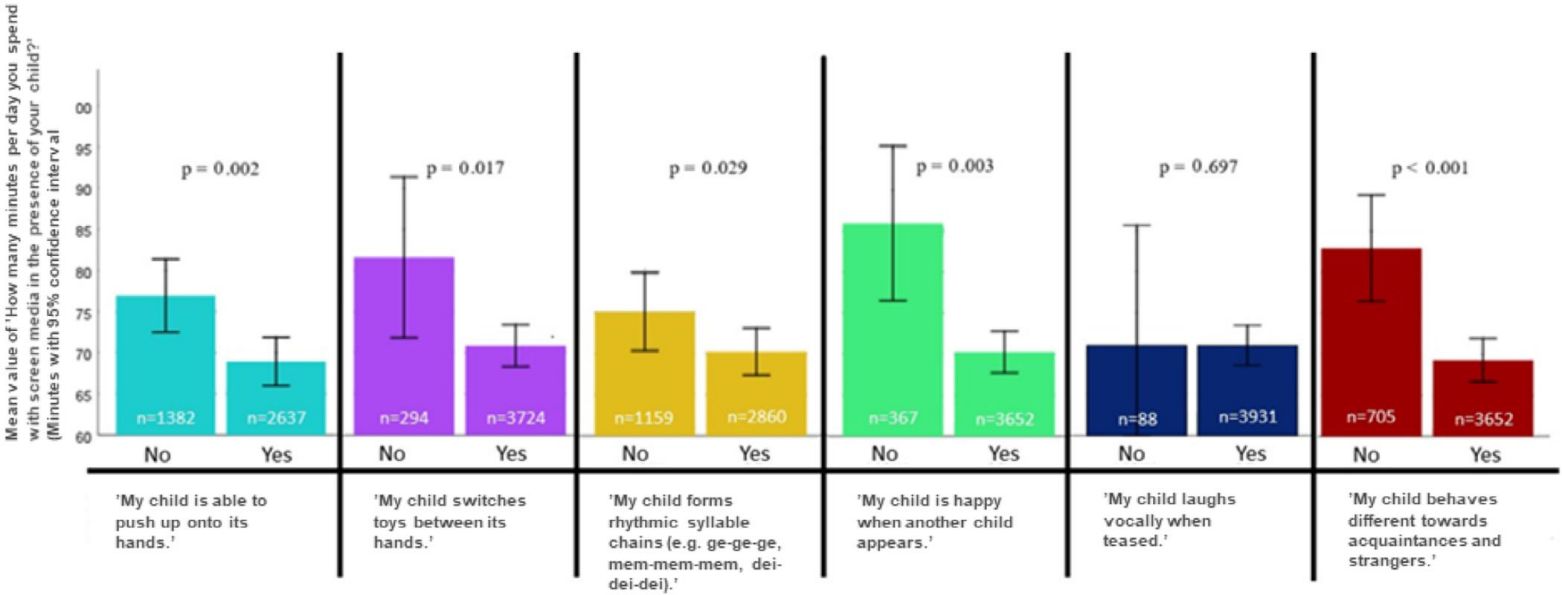
Parent screen time in the presence of the child and child development (the error bars represent the 95% confidence interval).

**FIGURE 3:**
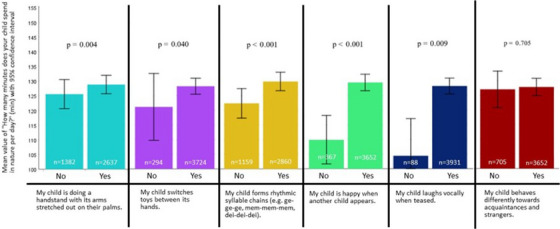
Time in nature and child development (the error bars represent the 95% confidence interval).

In the Pearson correlation, all developmental characteristics combined show a negative correlation with the PSTC (the higher the PSTC, the lower the developmental progress), *r* = −0.07, *p* < 0.001 (Figure 2). There are significant results with this test between the PSTC and children who engage in hand support with their arms stretched out, palms open, and those who do not (*U* = 1,711,527, *z* = −3.04, *p* = 0.002, *η* ^2^ = 0.002); children who switch toys between hands and those who do not (*U* = 501,460, *z* = −2.391, *p* = 0.017, *η* ^2^ = 0.001); children who form rhythmic syllable chains and those who do not (*U* = 1,579,955, *z* = −2.178, *p* = 0.029, *η* ^2^ = 0.001); children who behave differently toward acquaintances and strangers and those who do not (*U* = 1,049,280, *z* = −4.151, *p* < 0.001, *η* ^2^ = 0.004); children who express joy when another child appears and those who are not (*U* = 602,874, *z* = −3.011, *p* = 0.003, *η* ^2^ = 0.003). The Mann–Whitney *U* test also shows that children who spend more time in nature are significantly better positioned in the following developments (see also Figure 3)—engage in hand support (*U* = 1,891,294, *z* = 2.905, *p* = 0.004, *η* ^2^ = 0.002); switching toys between hands (*U* = 576,738, *z* = 2.005, *p* = 0.040, *η* ^2^ = 0.001); forming rhythmic syllable chains (*U* = 1,749,743, *z* = 3,913, *p* < 0.001, *η* ^2^ = 0.004); vocal laughter when being teased (*U* = 196,603, *z* = 2.627, *p* = 0.009, *η* ^2^ = 0.002); expression of joy when another child appears (*U* = 762,163, *z* = 5.256, *p* < 0.001, *η* ^2^ = 0.007). In the Pearson correlation, all developmental characteristics combined show a positive correlation with time spent in nature (the higher the time spent in nature, the better the developmental progress), *r* = 0.06, *p* < 0.001. The error bars in the graphs (Figures 2 and 3) represent the 95% confidence interval. The bars are large due to the high variance in the data. No information regarding screen time or time spent in nature was excluded as invalid, which means that even extreme values (i.e., several hours) remain in the dataset. This increases the variance and consequently the width of the confidence interval. Since no values were excluded, but these high values can still be considered outliers (i.e., outliers can significantly distort the mean and, in turn, the result of the *t*‐test), we chose not to use a *t*‐test for comparison. Instead, we employed the nonparametric Mann–Whitney *U* test.

## 5 Discussion

While the detrimental effects of screen time in early childhood have been fairly well documented (Deutsche Gesellschaft für Kinder‐ und Jugendmedizin e.V. DGKJ 2023; Hutton et al. 2020, 2022; Law et al. 2023; Li et al. 2020; Madigan et al. 2020; Schwarzer et al. 2022; Yang et al. 2020), the data presented here suggests that PSTC may also pose a risk. At worst, it can result in neglect of the child due to the use of screen media (Krasnova et al. 2015). The impact of reduced parental interaction and response to children's needs has been documented (Tronick et al. 1978), including that triggered by screen media (Tidemann and Melinder 2022).

### 5.1 Socio–demographics

In 2021, a total of 11.6 million families (with at least one child under the age of 18) lived in Germany, of which 23.3% of parents had a lower secondary school leaving certificate, 26.9% an intermediate secondary school leaving certificate, 39.6% a technical college or university entrance qualification, 4.2% another qualification, and 5.7% no qualification (Statistisches Bundesamt 2022). Data from the Federal Education Report showed that 52.8% of 25‐ to 29‐year‐olds from German households had a university entrance qualification (Statistisches Bundesamt 2021). In younger age groups, the proportion of people with a university entrance qualification increases, as does the proportion of women with a university entrance qualification, which in 2018 was 56% compared to just under 50% of men in the 25 to 29 age group, and a lower secondary school leaving certificate is less common (15.5%) (Statistisches Bundesamt 2021). Since the majority of participants in the “Screen‐free till 3” study are female (95.0%) and all are participating parents of infants, the proportion of participants in the “Screen‐free till 3” study with a university entrance qualification (61.8%) is close to the nationwide proportion. 9.5% of participants in the “Screen‐free till 3” study stated that their child had a migration background. In 2021, there were 785,000 families in Germany with the youngest child under the age of 1, of which 350,000 (44.6%) had a migration background (Statistisches Bundesamt 2022). This large difference may be due to selection bias with regard to the German‐language questionnaires. In this “Screen‐free till 3” study, 3.1% of parents are single parents; in contrast, according to the German Federal Statistical Office, there were 2.61 million (6.2%) single parents (children under 18) living in Germany in 2021, 82.3% of whom were mothers and 17.7% fathers. In the age group under 25 to 55 years, 1.79 million (4.26%) mothers (85.1%) and fathers (14.9%) living in Germany were single parents (Statistisches Bundesamt 2022). Considering that the “Screen‐free till 3” study is concerned with parents of infants aged 5 to 6 months and that the parents are most likely to be under 55 years of age, this is very close to the data from the Federal Statistical Office. Overall, the demographic data show a high degree of representativeness for the German population.

### 5.2 Problematic Internet Use

According to a survey conducted by the German Federal Center for Health Education (BZgA) in 2019, the prevalence of problematic internet use according to the CIUS (long version) is 5.1% for women and 3.2% for men among young adults aged 18 to 25 (Orth et al. 2020). In comparison, 16.4% of children and adolescents (10–17 years) met the ICD‐11 criteria for risky social media use and 6.3% for pathological social media use in a Forsa survey conducted by the German Center for Addiction Research in Childhood and Adolescence (DZSKJ) and the statutory health insurance in Germany (DAK‐Gesundheit) in June 2022, which has doubled compared to the prevalence in 2019 (DAK Gesundheit, 2023). There has, therefore, been a significant increase in the prevalence of risky and pathological use of the internet and also the use of screen media. Based on our data, problematic use of the internet by parents can be considered a risk factor for child development. In the group comparison, higher CIUS values of the parents were associated with higher screen time of the children, which in turn indicated a significant negative influence on the developmental characteristics of the children. The fact that 30.9% of fathers and only 16.7% of mothers reached the CIUS threshold is presumably due to the fact that mothers are still primarily occupied with caring for very young children.

### 5.3 Development

Statistical analysis has shown risk factors that can affect motor, language, and social/emotional development. This is primarily the PSTC with the following developmental steps: hand support, syllable strings, different behavior toward acquaintances and strangers, joy at the appearance of another child, switching toys between hands. The developmental areas affected may be precursors of the effects of dysregulated screen media use described in the literature. However, they also relate to the issue of healthy attachment, which is fundamental to a child's healthy development and includes the ability to show stranger anxiety, that is, the ability to show different behavior toward acquaintances and strangers. There are also indications of a risk potential in the case of problematic internet use by parents. This concerns the developmental steps joy at the appearance of another child and switching toys between hands. Considerations for monitoring the study population include forming and tracking defined risk groups (e.g., children with more than 1‐h of screen time), as well as monitoring extreme cases. The reported results are in line with literature. Previous research shows that high media usage in children is related to poorer social–emotional skills, while more frequent parent–child interactions are associated with better body motor and social–emotional skills (Schwarzer et al. 2022).

There is a positive correlation between time spent in nature and children's development, which is in agreement with previously published results. Outdoor play may mitigate the association between higher screen time and suboptimal neurodevelopment, and nature may be an underutilized public health resource for child psychological well‐being (Oswald et al. 2020; Sugiyama et al. 2023). Future follow‐up data of these children until the age of at least 3 years may reveal whether time spent in nature can also be seen as a kind of protective factor. An analysis carried out using logistic regression between time in front of the screen, time in nature, and children's development already suggests this at this early age, but was only very weak or not significant at this point. Screen usage is associated with slightly worse overall outcomes, while spending time in nature is linked to slightly better outcomes.

### 5.4 Limitations

As in all surveys, a bias due to social desirability must be assumed for the questions on screen media use (Morsbach and Prinz 2006). Given that the answers are anonymous, we have no reason to assume a large degree of social desirability bias. Studies on screen time estimation show both overestimation (Ohme et al. 2021) and underestimation (Hodes and Thomas 2021). We found no studies on the accuracy of estimation of parental screen time in the presence of toddlers, nor on the accuracy of the screen‐time estimation in toddlers. Given that many families have several screen devices, these issues are not easy to investigate, especially with regards to parental screen time in the presence of the child, as even eye‐tracking or face‐tracking cannot assess whether the child is near. A further limitation is that although most parents now have access to mobile phones, they could only take part in the questionnaire when the pediatrician services they used offered the MPP app and they had downloaded and used this app. The sociodemographic deviations of the sample from the average young mother were small and mainly concerned the migration background (Statistisches Bundesamt 2022). As the intervention materials and the survey are currently only available in German, it is planned to extend them to other languages in the future. The present paper presents a straightforward analysis of the data. More intricate methods, such as adjusted analyses that encompass child and family factors, could be applied in further publications. These limitations limit the generalizability of any conclusions derived from the data. The study cannot make any statement about a potentially negative influence of screen time before this measurement point, for example, due to screen media use during pregnancy and the first months of the children`s life (Birks et al. 2017; Schwarz et al. 2021).

## 6 Conclusion

The evaluation revealed a collective that is representative of German mothers and shows that screen media are used on a daily basis to a large extent. Some infants aged 5 to 7 months are already passively and actively exposed to screen media, contrary to national recommendations. Overall, this first survey suggests that high parental screen media use is linked to higher screen media use in children. The association between screen time and development on the one hand and nature and development on the other hand was significant but small in these children, with a median age of 5.5 months. The follow‐up data will show whether these effects are linked with trends in development trajectories.

Parental information and support to prevent excessive screen media use will be an increasingly important issue in the future. Society as a whole must be made aware of the importance of this topic. Families are bonding communities in which babies gain existential learning experiences primarily from other people and not from screens. In addition, time spent in nature could be a protective factor for developmental inhibitions due to screen media in early childhood.

## Disclosure

No material from other sources was used.

## Ethics Statement

All surveys and evaluations described were conducted with the approval of the responsible ethics committee, in accordance with national law and in accordance with the Declaration of Helsinki of 1975 (as amended and revised). The study has received a positive vote (No. 193/2020 dated October 15, 2020) from the Ethics Committee of Witten/Herdecke University.

## Conflicts of Interest

The authors declare no conflicts of interest.

## Data Availability

The data that support the findings of this study are available from the corresponding author upon reasonable request.
